# Titanium elastic nailing in pediatric femoral diaphyseal fractures in the age group of 6-15 years mid-term and long-term outcomes

**DOI:** 10.12669/pjms.346.16297

**Published:** 2018

**Authors:** Kemal Kayaokay, Kemal Aktuglu

**Affiliations:** 1 *Dr. Kemal Kayaokay, Department of Orthopaedics and Traumatology, Siverek State Hospital, Sanliurfa, Turkey*; 2 *Dr. Kemal Aktuglu, Department of Orthopaedics and Traumatology, Ege University Hospital, 35070 Bornova-Izmir, Turkey*

**Keywords:** Elastic nail, Pediatric femur fracture, limb length discrepancy, Flynn’s criteria

## Abstract

**Objective:**

To evaluate the effectiveness of Titanium Elastic Nailing (TEN) used in the surgical treatment of pediatric femoral shaft fractures and the effect of the complications to the outcome. Another objective was to assess the changing of Limb Length Discrepancy (LLD) and angulation degree with prolonged follow-up time and to evaluate whether the patient had a functional problem because of this situation.

**Methods:**

Thirty children between the ages of 6 and 15 who had femur shaft fractures were evaluated. The times of operation, ambulation, bone union and follow-up were recorded. Post-operative complications were evaluated between 1996-2016 with at least 24 Months follow up.

**Results:**

The mean follow-up was 52.5 ± 49.0 months (range 24-240). The mean varus angulation was 3.2 ± 5.1 degrees. The length of the fracture side was approximately 0.71 ± 0.58 cm (range 0-2.09 cm) longer than the intact side. There were eight patients with LLD of 1-2 cm. There was no statistically significant relationship between the type, location, and age of fracture of the LLD (P> 0.05). It was evaluated according to Flynn’s criteria. According to this, 12 (40%) of the patients’ results were excellent, 14 (46.7%) were good, and four were poor.

**Conclusion:**

TEN is an effective, easy, fast treatment method and has minimal complications for the treatment of femoral shaft fractures in childhood. Most complications can be reduced by performing basic principles and technical directions. Although LLD is a common complication of childhood femur fractures, the disease does not present a functional problem in daily life.

## INTRODUCTION

Pediatric femur fractures are common pediatric orthopedic injuries that require hospitalization.^1^ It accounts for about 1.6% of all bone fractures in children. It is more common in males than females (2.6: 1).^2^ Although pediatric femur fractures cause serious function loss in a short time, they can be treated successfully. There are many different options in children for treatment of femur fractures, such as spica casting, post-traction spica casting, Open Reduction Internal Fixation (ORIF), external fixation and Intramedullary (IM) nailing.^3^-^6^ In the past, these fractures were treated non-operatively with traditional treatment methods.^7^ Surgical treatment tendency has been increased due to improved surgical treatment options and implants in the last 30 years. It is important to return the child to the family environment as soon as possible, and to reduce the cost of care for the patient. 8,9 This will reduce the psychological negative effects of the child, as well as it will benefit the family and the child as social and economic.^10^,^11^

Elastic Stable Intramedullary Nailing (ESIN) is a common treatment option for pediatric femur fractures and has many advantages as compared with other treatment methods. It was first introduced in 1982 by the Nancy team in France under the name of Embrochage Centro Medullaire Elastique Stabile (ECMES).^11^ Osteosynthesis with the intramedullary elastic nail is performed by symmetrically inserting of the nails into the bone and fixation of the opposite metaphysis.^1^,^12^ It has begun to be preferred because of the small incision, less blood loss, no damage on epiphyseal of trochanter major, and increased interest in surgery. Although there is a consensus on the treatment of femur fractures in children aged five and below, various treatment modalities can be applied to children between 6-11 years old and children over 12 years old. Our study included femur fractures in the children who had weights less than 50 kg and between 6-15 years of age.

**Table-I:** Characteristics of patients.

		n	%
AO classification	A1	12	40.0%
	A2	5	16.7%
	A3	9	30.0%
	B1	2	6.7%
	B2	1	3.3%
	C1	1	3.3%
Fracture region	Proximal	8	26.7%
	Middle	21	70.0%
	Distal	1	3.3%
Side	Left	16	53.3%
	Right	14	46.7%
Gender
	Female	15	50.0%
	Male	15	50.0%

Age		Average±SS.	Median (Max. / Min.)

Cause of Trauma
	Female	9.27±2.79	14/6
	Male	8.00±2.70	15/6
	Total	8.63±2.74	
	In-vehicle accident	15	50.0%
	Vehicle off accident	6	20.0%
	Fall from high	6	20.0%
	Bicycle accident	3	10%

There is little data on mid-term and long-term follow-up of femur fractures that are seen in children aged 6-15 years. The purpose of this study was to evaluate the effectiveness of TEN (Titanium Elastic Nailing) used in the surgical treatment of pediatric femoral shaft fractures and the effect of the complications to the outcome. Another aim was to assess the changing of Limb Length Discrepancy (LLD) and angulation degree with prolonged follow-up time and to evaluate whether the patient had a functional problem because of this situation.

## METHODS

Patients with pediatric femur fractures who were treated with TEN and were followed-up for at least 24 months between 1996 and 2016 were evaluated retrospectively. Thirty patients were included who had regular follow-up and had orthoroentgenogram at the last follow-up. All the patients had closed femoral shaft fractures. Patients were between 6 and 15 years of age. Patients with open fractures, pathological fractures, metabolic bone disease, head trauma, and neurological deficit were excluded from the study. After the patients were evaluated, they were treated by closed reduction under fluoroscopy and internal fixation with titanium elastic nail (TEN). After osteosynthesis, the patient was treated with long leg cast. Two weeks after the operation, the leg molds were created and sutures were removed. Parameters such as pain, presence of soft tissue irritation due to nail, any infection, fracture healing, the range of motion were evaluated in the outpatient clinic controls. The time of union was evaluated as weeks by using x-ray. In addition, at the last follow-up, an orthoroentgenogram was performed to check whether there was limb length discrepancy. Pain, malalignment, LLD, and complications were reported and classified according to Flynn’s criteria. The study was approved by the Ege University Local ethics Committee (Approval No: 18-7.1 / 118).

Statistical analyzes were performed using IBM® SPSS® 25 (NY, USA) software. The normal distribution suitability of the variables was examined using analytical methods (Kolmogorov-Smirnov / Shapiro-Wilk tests). Descriptive statistics; mean ± standard deviation, median (IQR), minimum and maximum values. Frequency and percentage values were given for intermittent (categorical) variables. In the comparison of independent groups between continuous variables, the t test was analyzed in the independent groups in the variables with normal distribution, and Mann-Whitney U test in the normal non-distributed case. Statistical significance was considered when the p-value was below 0.05.

## RESULTS

Between 1996 and 2016, 30 patients with femur fracture were evaluated who had regular follow-up and had orthoroentgenogram at the last follow-up. All fractures were treated with two elastic nails inserted from distal metaphyseal retrogradely. Fifteen (50%) of the patients were female and 15 (50%) were male. The mean age was 8.63 (range 6 to 15). The mean follow-up was 52.5 ± 49.0 months (range 24-240). Patient demographics and associated injuries are shown in Table-I. Motor vehicle accident was the most common cause of femoral fractures in our study. Fifteen (50%) patients experienced in vehicle accidents and six (20%) patients had femoral fractures after a vehicle off accident. In Six patients, the fractures occurred after falling down from the high and three patients experienced bicycle accidents. Twenty one fractures (70%) were in the middle third of the femur, eight fractures were in the upper third of the femur, and one fracture was in the lower third of the femur located.

The mean time of taking operation after hospitalization was 2.33 days (1-6 days). In two patients, the fracture site was opened with a small incision to allow intraoperative reduction. The mean duration of operation was 54.5 minutes (range 30-72 minutes). All patients underwent long leg splint after the operation. In the postoperative period, cast and sutures were removed in the second week. Coronal and sagittal plane radiographs were taken to determine patient’s to evaluate union and weight bearing status. Patients’ mean weight bearing time was 6.3 weeks (4-8 weeks). All fractures were healed. The mean union time was 9.2 ± 2.2 weeks (range 6 to 15). Delayed union and nonunion were not seen. Irritation at the insertion site was present in two (1.5%) of the patients. No infection was detected in patients. Radiographically, coronal and sagittal planar diaphyseal angulation was measured. Angulation was seen in 16 patients. The most common type of angulation was varus angulation. Eleven patients had varus angulation. The mean varus angulation was 3.2 ± 5.1 degrees. The angulation was between 5-10 degrees in 10 patients and the angulation was over 10 degrees in three patients. Limb length discrepancy was measured by orthoroentgenogram. Fifteen patients had LLD. The length of the fracture side was approximately 0.71 ± 0.58 cm (range 0-2.09 cm) longer than the intact side. There were 8 patients with LLD of 1-2 cm. The discrepancy was over 2 cm in one patient. Measurements were made from this patient’s 240-month radiographs. There was no statistically significant relationship between the type, location, and age of fracture of the LLD. (P> 0.05) Patient follow up findings are shown in Table-II. In addition, patients were evaluated according to Flynn’s criteria. According to this, 12 (40%) of the patients’ results were excellent, 14 (46.7%) were good, and four were poor. Patients with LLD were not aware of the elongation and have not reported any functional complaints that affected daily life. Flynn’s Score was compared with fracture sites, side, fracture types and there was no statistically significant difference (p = 0,152).

**Table-II:** Follow up findings.

	N	Mean±SD.	Median (Max. / Min.)
Operation time (minutes)	30	54,5±11,7	53,5 (72 / 30)
Full weight bearing (weeks)	30	6,3±0,8	6 (8 / 4)
Radiological union (weeks)	30	9,2±2,2	9 (15 / 6)
Follow-up (months)	30	52,5±49,0	35 (240 / 24)
Post-op varus angulation- degrees	30	3,2±5,1	0 (15 / 0)
Valgus angulation - degrees	30	0,53±1,40	0 (5 / 0)
Anterior- Posterior angulation	30	2,4±5,2	0 (25 / 0)
Leg-length discrepancy	30	0,71±0,58	0,15 (2,09 / 0,00)

SD.: Standart deviation -Max. Maximum - Min. Minimum

## DISCUSSION

Pediatric femoral shaft fractures have been treated conservatively in the past.^7^ Conservative treatment methods are used as standard in the treatment of children under 5 years.^13^ There are different treatment options for femur fractures in children who are between 6-15 years old. The choice of surgical treatment in this age group depends on the age of the patient, localization of the fracture, the experience of the surgeon. TEN is the most commonly used treatment method in patients who are between 6 and 15 years old.^14^ In this age group, it is important that the patients move during treatment independently. The return of the child to the social environment is important in terms of psychosocial development. Taking time off work during the course of care will cause financial loss for family.^15^ Considering these factors, the importance of this treatment method is increasing. In our clinic, the patients with femur fracture who are in this age group are treated with TEN. In this treatment described by the ’Nancy team’, nail is selected by measuring 40% of the narrowest diameter of the medullary canal.^12^ Elastic intramedullary nail treatment works by balancing the forces between the two opposing flexible implants. TEN is an elastic stable intramedullary nail that works on the principle of three-point fixation by resisting distraction and compression forces.^12^,^14^

No nonunion or delayed union was seen in our study. The mean union time was 9.2 ± 2.2 weeks (range 6 to 15). Acceptable angulation in pediatric fractures have been reported by many authors. Cadman and Neer accept that up to 15 degrees of angulation is maximum; Buehler et al. accept that less than or equal to 20 degrees on the coronal plane and less than 30 degrees on the sagittal plane are appropriate.^16^ In our study, two patients had 10 degrees of angulation on the coronal plane, and one patient had more than 10 degrees of angulation on the coronal and sagittal planes. Flynn et al. reported that lower than 10 degrees of angulation is at an acceptable limit.^14^ According to Flynn’s criteria, we had three patients with poor results because of the angulation. It was seen that the angulation decreased in time. We thought that this complication can be decreased by surgical experience, appropriate technique, and careful follow-up.^17^,^18^

LLD is a known and unavoidable complication of elastic intramedullary nailing that is performed to pediatric femur shaft fractures. LLD is associated with age, sex, fracture type, fracture type. Overgrowth is more common in patients aged 2-10.^19^ In 1921, Truesdell described that the fracture healing process stimulates bone growth in femoral shaft fractures in children.^20^ Staheli reported that over growth was greatest in the proximal of the femur.^21^ On the contrary Henry reported that overgrowth was greatest in the distal of the femur.^22^

In our study, bone length increased in 15 patients. In the patients with LLD, five were proximal, nine were middle, and one was distal femur. There was no statistically significant difference between the fracture site and LLD (p 0.156). As the follow-up period prolonged, the LLD increased. There was a statistically significant correlation between the follow-up period and the amount of elongation (rS = 0,622; p = 0,0001). (Spearman) According to Flynn’s criteria, the most common complication which caused satisfactory and poor results was the angulation. Similar results were obtained with recent studies in the literature.^18^

According to Flynn’s criterias, poor results were seen in our study in four patients. Three of these patients had angular deformity and one patient had LLD of 2.09 cm. It was remarkable that there were no functional complaints affecting the daily life from patients who had poor results.

Elastic Intramedullary Nailing is a successful method of treating childhood femoral shaft fractures. It is a simple, rapid, and less intraoperative complications, depending on the experience of the surgeon. Early mobilization and short-time hospitalization will be beneficial for the children and their families in many ways. Complications will decrease by surgical experience.

## CONCLUSION

TEN is an effective, easy, fast treatment method and has minimal complications for the treatment of femoral shaft fractures in childhood. Most complications can be reduced by performing basic principles and technical directions. Although LLD is a common complication of childhood femur fractures, it doesn’t cause a functional problem in daily life.

### Authors Contribution

**KK:** Conceived, designed and did statistical analysis & editing of manuscript.

**KK:** Did data collection and manuscript writing.

**KA:** Did review and final approval of manuscript.
